# Investigation of Metabolites in Feces and Plasma Associated with the Number of Piglets Weaned per Sow per Year

**DOI:** 10.3390/metabo15110683

**Published:** 2025-10-22

**Authors:** Takamitsu Tsukahara, Hiroto Miura, Takahiro Kawase, Shu Yoshimura, Yoshihiro Mizukami, Yoshihiro Yahara, Kikuto Fukuta, Ryo Inoue

**Affiliations:** 1 Kyoto Institute of Nutrition & Pathology, Kyoto 610-0231, Japan; tsukahara@kyoto-inp.co.jp (T.T.); kawase@kyoto-inp.co.jp (T.K.); 2 Laboratory of Animal Science, Department of Applied Biological Sciences, Faculty of Agriculture, Setsunan University, Hirakata 573-0101, Japan; hiroto.miura@setsunan.ac.jp; 3 Laboratory of Animal Function and Nutrition, Research Faculty of Agriculture, Hokkaido University, Sapporo 060-8589, Japan; 4 Marubeni Nisshin Feed Co., Ltd., Nasushiobara 329-2763, Japan; yoshimura-s@mn-feed.com (S.Y.); yahara@tonjikyo.or.jp (Y.Y.); 5 Akabane Animal Clinic, Tahara 441-3502, Japan; water_up@hotmail.co.jp; 6 Toyohashi Feed Mills, Shinshiro 441-1346, Japan; k-hukuta@toyohashi-shiryo.co.jp

**Keywords:** glucuronyl- or sulpho-conjugate of intestinal putrefactive metabolites, inflammation, reproductive performance, short-chain fatty acid, sow, trimethylamine *N*-oxide, tumor necrosis factor-alpha

## Abstract

**Background**: Sow reproductive performance is a critical parameter for the productivity of commercial pig farms. Gut microbiota is associated with performance in sow reproduction. At least, under healthy conditions, microbial metabolites from the gut microbiota are considered major contributors to host physiological regulation and productivity. However, information on the differences in gut-derived metabolites related to the sow reproductive performance remain scarce. Our aim was to investigate the relationship between the reproductive performance and microbial metabolite levels in sow’s feces and plasma. **Methods**: We selected four commercial farms: two with high- (group H) and two with low-reproductive performance (group L). Sows had their feces and blood collected. **Results**: Except for the iso-butyrate concentration, fecal short-chain fatty acid concentrations remained unchanged between groups. Among intestinal putrefactive metabolites, the indole concentration was higher (*p* < 0.05) in group H. The concentrations of plasma metabolites *p*-cresyl sulfate, *p*-cresyl glucuronide and trimethylamine *N*-oxide (TMAO) were higher (*p* < 0.05) in group L than in group H, while the opposite was true for the acetate concentration (*p* < 0.05). Among plasma biochemicals, tumor necrosis factor (TNF)-alpha and potassium concentrations were higher (*p* < 0.05) in group L. **Conclusions**: Blood metabolites, especially gut microbiota-derived metabolites, seemed to be associated with the performance related to sow reproduction. Particularly, harmful metabolites such as *p*-cresyl glucuronide, *p*-cresyl sulfate and TMAO were of importance, because they are potentially inflammation factors. In fact, TNF-alpha was stimulated in group L. According to our results, we estimated that *p*-cresyl glucuronide, *p*-cresyl sulfate, TMAO and TNF-alpha could be useful physiological indicators to understand sow reproductive performance.

## 1. Introduction

Sow reproductive performance is a crucial parameter for the productivity of commercial pig farms. It is determined by sow fertility and prolificacy, which are reflected in benchmarking measurements such as the number of piglets weaned per sow per year [1]. The factors influencing sow reproductive performance have been investigated from various perspectives. For example, Thacker and Bilkei [2] reported that weight loss during lactation affects reproductive outcomes such as the weaning-to-service interval, which is one of the indicators for sow fertility. In a study by Chen et al. [3], compared with those with a normal backfat thickness of 17 mm, sows with a backfat thickness greater than 21 mm had significantly lower numbers of piglets born alive, an indicator closely related to prolificacy. Sows also exhibited elevated levels of the plasma inflammatory cytokine TNF-α and IL-6.

In recent years, several studies have suggested that the gut microbiota is also associated with sow reproductive metrics [4]. We also previously demonstrated that the gut microbiotas of sows displayed distinct characteristics depending on individual farms and differed according to their reproductive performance (i.e., the number of piglets weaned per sow per year) in those farms [5,6]. However, studies investigating the differences in blood metabolites related to the sow reproductive performance remain scarce. In particular, metabolites derived from the gut microbiota have not been evaluated in this context except for the short-chain fatty acids (SCFAs).

Gut microbiota influences host physiology primarily through two pathways: (1) by translocation, and (2) by the microbial metabolites [7]. The translocation of gut bacteria or their components is strongly associated with leaky gut conditions [7], whereas microbial metabolites are absorbed by the host via active transport or passive diffusion [8,9]. Therefore, under healthy gut conditions (i.e., without experiencing leaky gut), bacterial translocation rarely occurs, and the microbial metabolites are considered major contributors to the host physiological regulation and productivity.

Among the microbial metabolites, SCFAs are the most well-known for their beneficial effects on host animals. SCFAs not only provide approximately 25% of the metabolic energy for pigs [10] but also regulate various physiological functions, including gut motility, anti-inflammatory responses, and stress reduction [9,11]. In contrast, potentially harmful compounds, collectively referred to as intestinal putrefactive metabolites (IPMs), such as phenol, indole, skatole, and *p*-cresol, are also produced by the gut bacteria [12,13]. These metabolites have been suggested to negatively affect productivity [14]. After absorption, IPMs are rapidly metabolized into glucuronide or sulfate conjugates in the intestinal tissue or liver [15]. However, these conjugates are not fully detoxified, and retain toxicity to the host [15,16]. Trimethylamine (TMA), another microbial metabolite derived from choline and _L_-carnitine, is absorbed and further metabolized to trimethylamine-*N*-oxide (TMAO) in the liver [17]. TMAO is suspected to negatively affect host health [18] and may also be linked to reduced reproductive performance of breeding sows.

In addition to gut-derived metabolites, plasma free amino acids (AAs), which reflect protein catabolism and/or dietary protein derivation, are well-established physiological indicators for various diseases in humans. However, their relationship with the reproductive performance of breeding sows remains poorly understood.

The aim of the present study was to investigate the relations between the sow reproductive performance and plasma metabolite levels. To complement this, gut microbiota-derived metabolites in the feces were also evaluated. We selected four commercial farms: two with high reproductive performance (H1 and H2) and two with low reproductive performance (L1 and L2). At each farm, fecal and blood samples from 7 to 10 healthy sows were collected.

## 2. Materials and Methods

### 2.1. Description of Farms and Sampling Procedures

Farms H1, H2, L1 and L2 (breeding line; Landrace × Large White or Large White × Landrace) were introduced to the current study. The numbers of piglets weaned per sow per year in the respective farms are summarized in Table 1. In our previous report [5], many parameters related to reproductive performance, such as farrowing rate, farrowing/sow/year, number of piglets born/litter, and number of piglets born alive/litter, were positively and significantly correlated with the number of piglets weaned per sow per year. Thus, the number of piglets weaned per sow per year was selected as the representative parameter for reproductive performance of each farm. Sows in H1 and H2 farms were allocated to group H, and those in L1 and L2 to group L. The sampling size of the number of sows was determined based on the data from our previous study [5].

Fresh feces were sampled from 7 or 10 healthy-, multiparous- and late-pregnancy sows (2nd–5th parities; L2, n = 7; other farms, n = 10), as previously described [5,6]. Sows with a body condition score within 3–3.5 were selected. Heparinized blood samples were also collected from the jugular veins of the same sows. Feces and blood were sampled between the 10th and the 14th week of pregnancy. After collection, blood and feces were immediately cooled on ice, and transported to the Kyoto Institute of Nutrition & Pathology (Kyoto, Japan) within 24 h. Plasma was separated from the blood samples by centrifugation (3000× *g*, 10 min, 4 °C), and then kept at −80 °C until further analysis. Feces were gently homogenized and stored at −80 °C until analysis. The analyses were performed under blinded conditions.

### 2.2. Analysis of Bacterial Metabolites in Feces

Ion-exclusion high-performance liquid chromatography (HPLC) was used to quantify fecal organic acids: formate, acetate, lactate, propionate, succinate, iso-butyrate, n-butyrate, iso-valerate, n-valerate. Briefly, 0.3 g of feces was homogenized in 0.6 mL distilled water, mixed with 90 μL of 12% perchloric acid (*v*/*v*), and centrifuged (13,000× *g*, 10 min, 4 °C). The supernatant was passed through 0.45 μm cellulose acetate filters (Cosmonice Filter W, Nakalai Tesque, Kyoto, Japan) and transferred to vials. Samples were injected into an HPLC system equipped with a SIL-10 autoinjector (Shimadzu, Kyoto, Japan). Organic acids were separated using two serial organic acid columns (Shim-pack SCR-102H, Shimadzu) with a guard column (SCR-102HG, Shimadzu) at 45 °C under isocratic conditions (0.8 mL/min) with 5 mmol/L *p*-toluene sulfonic acid solution, delivered by an LC-10ADvp pump (Shimadzu) with a degasser (DGU-12A, Shimadzu). Detection was performed using an electronic conductivity detector (CDD-10AVP, Shimadzu) after post-column dissociation (0.8 mL/min) with 5 mmol/L *p*-toluene sulfonic acid, 20 mmol/L bis-Tris, and 100 μmol/L EDTA, delivered by an LC-10ADvp pump. Quantification was controlled by a CBM-20A system controller (Shimadzu).

Fecal IPMs (phenol, *p*-cresol, indole and skatole) were measured by gas chromatography–mass spectrometry (GCMS-QP2010 Ultra; Shimadzu) equipped with an autosampler (AOC-5000; Shimadzu). Hundred milligrams of feces was thawed and placed in 2 mL screw-cap tubes (Watson Co., Ltd., Tokyo, Japan) with a 5.5 mm stainless steel ball (TOMY, Tokyo, Japan). Each sample was mixed with 0.9 mL methanol and 10 μL of internal standard solution [1 mmol/L phenol-d_5_ in methanol (Sigma-Aldrich Japan, Tokyo, Japan)] and homogenized at 3000 r.p.m. for 30 s with Micro Smash MS-100 (TOMY, Tokyo, Japan). After centrifugation at 15,000× *g* for 5 min (4 °C), the supernatant was transferred to a 3.5 mL screw-cap cryotube (TPP Techno Plastic Products, Trasadingen, Switzerland). The remaining pellet was extracted again with 1 mL methanol following the same procedure, and this was repeated twice. The combined supernatant (about 2.9 mL) was pooled for subsequent analysis. An InertCap5 column (30 m × 0.25 mm I.D., 0.25 μm; GL Sciences Inc., Tokyo, Japan) was used for chromatographic separation with helium as the carrier gas at 48.1 mL/s. The oven temperature was held at 40 °C for 2 min, then ramped to 320 °C at 10 °C/min and maintained for 10 min, giving a total run time of 40 min. The mass spectrometer operated in SIM mode targeting *m/z* 117.0 (indole), 108.0 (*p*-cresol), 94.0 (phenol), 99.0 (phenol-d_5_), and 131.0 (skatole). Samples were injected in split mode (10:1) with a 1 μL volume at 250 °C. Chemical ionization was performed with an ion source temperature of 200 °C and interface temperature of 250 °C. Concentrations were calculated using peak area ratios relative to phenol-d_5_.

The concentration of fecal trimethylamine (TMA) was measured by an ultra-performance liquid chromatography tandem mass spectrometry (LC-MS/MS) system (Acquity TQD UPLC-MS/MS system; Waters, Milford, MA, USA). Thawed feces (300 mg) were transferred to 2.0 mL screw-cap tubes (Watson Co., Ltd., Tokyo, Japan) along with one 5.5ϕ stainless steel ball (TOMY) per tube. Three hundred microliters of trimethylamine-d_9_ hydrochloride (d_9_-TMA; 10 mmol/L; Toronto Research Chemicals, Toronto, Ontario, Canada) was added as an internal standard solution and the contents were vigorously mixed with the above-mentioned homogenizer (Micro Smash MS-100) on the same setting. The mixtures were centrifuged (15,000× *g*, 10 min, 4 °C) and the supernatants were collected and loaded onto ultrafiltration filters (Amicon Ultra 0.5 mL 1K, MerckMillipore Japan, Tokyo, Japan), and the samples were centrifuged again (14,000× *g*, 15 min, 4 °C). Supernatants were collected and used as samples for further analysis. Chromatographic separation was conducted with an Intrada Amino Acid column (3.0 μm × 100 mm × 3 mm; Imtakt, Kyoto, Japan). The mobile phase, delivered at a flow rate of 0.4 mL/min at 40 °C, was a gradient of solution A (0.1% formic acid–acetonitrile, Fujifilm Wako, Osaka, Japan) and solution B (acetonitrile/100 mM ammonium formate = 20/80, Fujifilm Wako). The gradient program was as follows: solution A was decreased from 75% to 0% over 10 min, followed by a 2 min hold at 0%. The total run time was 12 min. Detection was performed using a tandem quadrupole mass spectrometer (Waters) equipped with a Z-spray ion interface and controlled by Waters MassLynx software version 4.2 (Waters). Ionization was achieved in alternating electrospray positive-ion mode. Positive-ion settings were ion source temperature, 150 °C; capillary voltage, 2.5 kV; desolvation temperature, 480 °C; desolvation gas flow, 900 L/h; and cone gas flow, 55 L/h. Multiple-reaction monitoring (MRM) was conducted by analyzing product ions of deprotonated molecules from TMA and d_9_-TMA. The *m*/*z* values of TMA and d_9_-TMA were 60.3 (Q1) to 45.3 (Q3) and 69.3 (Q1) to 51.3 (Q3), respectively. Cone voltage and collision energy for both materials were set as 30 V and 10 V, respectively. The injection volume was 5 µL.

### 2.3. Analysis of Bacterial Metabolites in Plasma

A portion of plasma was used to analyze by GC-MS the concentrations of SCFAs such as acetate, propionate, iso-butyrate, n-butyrate, iso-valerate, n-valerate and caproate. The measurement procedure was the same as that previously described [19].

Another portion of plasma was used to analyze by LC-MS/MS the concentrations of glucuronyl- or sulpho-conjugates of IPMs (phenyl glucuronide, indoxyl glucuronide, *p*-cresyl glucuronide, phenyl sulfate, indoxyl sulfate and *p*-cresyl sulfate). The measurement procedure was same with that previously described [20].

Measurement of plasma trimethylamine *N*-oxide (TMAO) was also performed on an Acquity TQD UPLC-MS/MS system. Based on the method of Jia et al. [21], the procedure was as follows. Fifty microliters of plasma was transferred to 1.5 mL microtubes. A 10 μL aliquot of internal standard solution [10 mmol/L trimethylamine-d_9_ *N*-oxide (d_9_-TMAO); Cayman Chemical, Ann Arbor, MI, USA] was added and vortexed. Next, 50 µL of 0.4 mol/L perchloric acid was added and the mixtures were vortexed again. After cooling on ice for 30 min in a dark place, the samples were centrifuged (15,000× *g*, 15 min, 4 °C). The supernatants were filtered using 0.2 µm pore-size syringe filters (SY4PL-S; Advanced Microdevices Pvt. Ltd., Ambala Cantt, India) and transferred to vials. Chromatographic separation was conducted using an Inertsil HILIC column (3.0 μm × 150 mm × 2.1 mm; GL Sciences Inc.). Chromatography was carried out using a gradient of solution A (ultrapure water; Fujifilm Wako) and solution B (ultrapure acetonitrile; Fujifilm Wako) at 0.2 mL/min and 40 °C. The gradient started with 99% A for 3 min, decreased to 40% in 1 min, held for 2 min, then decreased to 0% in 2 min and held for 4 min (total run time: 12 min). Detection employed a Waters tandem quadrupole mass spectrometer with a Z-spray interface, controlled by MassLynx software. Ionization was performed in alternating electrospray positive mode with source temperature at 120 °C, capillary voltage at 3.0 kV, and desolvation temperature at 350 °C. Gas flows were 600 L/h (desolvation) and 50 L/h (cone). Multiple-reaction monitoring was used to detect product ions from TMAO and d_9_-TMAO. The *m*/*z* values of TMAO and d_9_-TMAO were 76.0 (Q1) to 59.2 (Q3) and 85.0 (Q1) to 68.3 (Q3), respectively. Cone voltage and collision energy for both materials were set as 30 V and 10 V, respectively. The injection volume was 5 µL.

### 2.4. Analysis of Plasma Biochemicals

Plasma biochemicals such as aspartate aminotransferase (AST), alanine aminotransferase (ALT), creatine phosphokinase (CPK), urea nitrogen (BUN), total cholesterol (Tcho), creatinine, sodium, calcium, chloride and potassium were measured at a commercial laboratory (Association of Kyoto Microbial Laboratory, Kyoto, Japan).

The tumor necrosis factor (TNF)-alpha concentration in plasma was measured with a commercial ELISA kit (Porcine TNF-alpha ELISA Kit Quantikine; R&D systems, Minneapolis, MN, USA), as per the manufacturer’s instructions.

Measurement of plasma AAs were also performed on an Acquity TQD UPLC-MS/MS system. An internal standard mixture was purchased from Taiyo Nippon Sanso Corporation (Tokyo, Japan), which contained 20 mM stable isotopic nuclei (^13^C and ^15^N) of constituent amino acids. Thawed plasma (100 µL) was transferred to 1.5 mL microtubes, then 5 µL of the internal standard solution (2 mmol/L) as well as 400 µL of ultrapure water were added, and the mixtures were vortexed. The solutions were loaded onto ultrafiltration filters (Amicon Ultra 0.5 mL 3K, MerckMillipore Japan) and centrifuged (14,000× *g*, 15 min, 4 °C). Supernatants were collected and used as samples for further analysis. Chromatographic separation was conducted with an Intrada Amino Acid column (3.0 μm × 100 mm × 3.0 mm; Imtakt). The mobile phase, delivered at a flow rate of 0.6 mL/min at 35 °C, was a gradient of solution A (acetonitrile/tetrahydrofuran/25 mM ammonium formate/formic acid = 9/75/16/0.3; Fujifilm Wako) and solution B (acetonitrile/100 mM ammonium formate = 20/80; Fujifilm Wako). The gradient started with 100% solution A for 3 min, decreased to 83% over 6 min, then dropped to 0% in 0.5 min and held for 6.5 min (total run time: 16 min). Analysis was performed using a Waters tandem quadrupole MS with a Z-spray interface, controlled by MassLynx software. Ionization used an alternating electrospray positive mode with source temperature at 150 °C, capillary voltage at 3.0 kV, and desolvation temperature at 450 °C. Gas flows were 850 L/h (desolvation) and 60 L/h (cone). Multiple-reaction monitoring detected product ions from amino acids, with details provided in Supplementary Table S1. The injection volume was 5 μL.

### 2.5. Statistical Analysis

To investigate the homoscedasticity, the parameters for the feces and plasma were analyzed with the F test. Based on the results, either Student’s or Welch’s *t*-test was applied to compare group differences using statcel 2, an add-on for Excel (OMS Publishing, Saitama, Japan). Spearman’s rank correlation coefficient was applied to evaluate the correlations between fecal and plasma metabolites concentrations.

All statistical values are presented as arithmetic means ± standard errors. Differences between means were considered statistically significant at *p* < 0.05.

## 3. Results

### 3.1. Bacterial Metabolites in Feces

Table 2 shows the results of the analysis of bacterial metabolites such as organic acid, putrefactive metabolites and TMA concentrations.

No significant differences were observed in acetate, propionate, n-butyrate, iso-valerate, or n-valerate concentration; however, iso-butyrate was higher in group L than in group H. The concentrations of phenol, *p*-cresol and skatole did not significantly differ between groups; however, the indole concentration was higher in group H than in group L. Succinate, lactate, formate and TMA were not detected or detected only in traces in all feces; therefore, we omitted these materials from the reported results.

### 3.2. Bacterial Metabolites in Plasma

The analysis results of bacterial metabolites such as SCFAs, glucuronyl- and sulpho-conjugates of IPMs and TMAO concentrations are shown in Table 3.

Acetate, phenyl glucuronide, *p*-cresyl glucuronide, *p*-cresyl sulfate and TMAO concentrations were higher in group L than in group H. iso-Butyrate, phenyl sulfate and indoxyl sulfate concentrations did not significantly differ between groups. As propionate, n-butyrate, n-valerate, iso-valerate, caproate and indoxyl glucuronide were not detected or detected only in one to four sows (total: 37 sows), we chose to omit these data from the reported results.

We further analyzed the correlation coefficients between the fecal and plasma metabolites (Supplementary Figure S1). No meaningful correlation suggesting direct association of fecal and plasma metabolites was observed.

### 3.3. Biochemical Composition of Plasma

The TNF-alpha concentration and the biochemical parameters in plasma are shown in Table 4. TNF-alpha and potassium concentrations were higher in group L than in group H. By contrast, ALT, AST, CPK, BUN, Tcho, creatinine, sodium, calcium and chloride concentrations did not significantly differ between groups.

Regarding the AA concentration, tryptophan, phenylalanine, glutamine, asparagine, histidine and lysine concentrations in plasma were higher in group H than in group L, while the proline concentration was lower in group H than in group L (Table 5). Other amino acids such as tyrosine, leucine, isoleucine, valine, methionine, glutamic acid, threonine, alanine, serine, glycine and arginine concentrations did not significantly differ between groups.

## 4. Discussion

### 4.1. Bacterial Metabolites

#### 4.1.1. Fecal Metabolites

In the present study, except for the iso-butyrate concentration, the fecal SCFA concentrations did not significantly change between groups (Table 2). In contrast, fecal concentrations of IPMs were relatively higher in group H than in group L, particularly the indole concentration, which was significantly higher (Table 2). Amino acids in the large intestine, including tryptophan and tyrosine, are the major sources of IPMs [12,22]. IPMs are metabolized from amino acids by the gut microbiota; a higher concentration of indole in the feces of sows in group H might be explained by two possible scenarios: (1) domination of tryptophan- and/or tyrosine-degrading bacteria in the gut of H sows, or (2) a lower absorption of indole from the large intestine. The plasma concentration of indole conjugate and indoxyl sulfate did not differ between groups (Table 3). Therefore, the latter may be the more plausible explanation for the higher concentration of indole in group H. However, this point should be further investigated in future studies, as conclusive evidence was not obtained in the present work.

#### 4.1.2. Plasma Metabolites

Absorbed SCFAs flow into the liver via the portal vein, and hepatocytes convert propionate and n-butyrate to metabolic energy [23]. Therefore, the concentrations of propionate and n-butyrate are found only in traces in the peripheral blood of pigs [24], as was the case in the present work. In contrast, acetate was clearly detected and the concentration was significantly lower in group H than in group L (Table 3). Acetate, derived mainly from the gut microbiota [25], is utilized by the brain and the skeletal muscles [26] as a source of energy. Therefore, we speculated that a lower concentration of acetate in group H was due to a more efficient acetate utilization as an energy source in the tissues of the sows. Nonetheless, the reason for this difference in the acetate concentration needs to be further investigated. The plasma concentration of iso-butyrate was higher in group L than in group H, yet this difference was not significant (Table 3). Branched-chain fatty acids are the metabolites of the gut microbiota produced mainly from branched-chain amino acids (bCAA) such as leucine, isoleucine and valine [27]. As plasma bCAA concentrations were not different between groups (Table 5), the absorption of bCAA in the small intestine was putatively not different between groups. In this context, a higher iso-butyrate concentration in group L could mean a higher production of iso-butyrate by bCAA-utilizing bacteria in the large intestine.

The plasma concentrations of IPMs conjugates such as *p*-cresyl glucuronide and sulfate were lower in group H than in group L (Table 3). All things considered, we speculated that IPM permeation was lower in group H than in group L. As mentioned above, premetallized IPMs are harmful and known to be uremic toxins. For example, *p*-cresyl sulfate is a pro-inflammatory material [15], and *p*-cresyl glucuronide induces cell stress in human renal tubule epithelial cells [28]. Therefore, these conjugated IPMs, which were found to be higher in group L, might be associated with the low reproductive performance of breeding sows.

While TMA were not detected in the feces, its metabolites, TMAO, were detected in the plasma and were significantly higher in group L than in group H (Table 3). The biological role of TMAO is still not fully understood [18,29]. However, it has been reported that TMAO could be a pro-inflammatory agent [30] and associated with several chronic disorders in humans [31]. By extension, it can be assumed that TMAO may also be associated with the lower productive performance of sows.

Some correlations between bacterial metabolites in feces and plasma were observed (Supplementary Figure S1). However, no biologically meaningful associations that would justify a detailed relationship between fecal bacterial metabolites and plasma biochemical parameters were identified. This may be due to the complex absorption and metabolic processes that occur between the gut and systemic circulation.

### 4.2. Plasma Biochemicals

Plasma TNF-alpha is one of the major inflammation markers in pigs [32] and many studies have suggested that high TNF-alpha levels result in lower productivity of sows, including reproductive performance [3,33,34]. TNF-alpha is elevated during pathogen infection or chronic inflammation mediated by various stresses such as heat stress [35]. In the present work, the plasma TNF-alpha concentration was significantly higher in group L than group H, though no obvious infections were observed in the sows (Table 4). An elevated level of plasma TNF-alpha may have resulted from the high concentrations of harmful bacterial metabolites such as *p*-cresyl sulfate, *p*-cresyl glucuronide and TMAO, which are pro-inflammatory agents.

In the present study, the concentration of potassium in plasma was higher in group L than in group H. A major risk factor for hyperkalemia is renal failure [36]. Creatinine, a marker of renal function, was not different between groups (Table 4). Nonetheless, *p*-cresyl sulfate and glucuronide are known to be uremic toxins [15]. Higher concentrations of these conjugates could induce renal malfunction, which in turn may result in hyperkalemia. By contrast, supplementation of potassium diformate (1.2% added to feed) to sows does not modify the productive performance of sows [37]. We believe that, in the present study, a higher concentration of potassium in plasma is unlikely to have signifivantly affected the sow reproductive performance. Therefore, a higher concentration of potassium in group L may be considered the result of a secondary reaction.

Tryptophan, lysine, phenylalanine, and histidine, which are categorized as essential amino acids, were significantly lower in the plasma of group L (Table 5). Among these AAs, the difference in tryptophan is most noteworthy. A decrease in tryptophan in plasma can be observed when the kynurenine pathway is activated due to chronic inflammation. Metabolizing tryptophan into kynurenine is initiated by the enzyme indoleamine 2,3-dioxygenase, which is upregulated by inflammatory cytokines like TNF-alpha [38]. Kynurenic acid, the resulting intermediate metabolite of this pathway, exerts anti-inflammatory properties [39]. Therefore, a lower tryptophan concentration in the plasma of group L is likely a homeostatic mechanism for the regulation of inflammation. In fact, previous work demonstrated depletion of plasma tryptophan in pigs following experimentally induced chronic lung inflammation [40] and a chronic immunological challenge from unhygienic housing conditions [41]. Regarding the non-essential AAs in plasma, lower levels of glutamine and asparagine in group L can be attributed to their increased consumption in response to inflammation. Although glutamine is nutritionally a non-essential AA, it becomes a conditionally essential AA during inflammation [42]. As discussed above, group L had a more inflammatory status than group H, and thus a need for glutamine may have been increased, which resulted in its reduction in the plasma pool. A similar scenario can explain the decrease in asparagine, because it can be converted from glutamine and become essential for immune functions [43]. The concentration of proline was higher in the plasma of group L than in that of group H. Taken together with the fact that plasma CPK tended to be higher (*p* < 0.1) in group L, the increase in plasma proline might have been due to skeletal muscle catabolism induced by inflammation. Orellana et al. [44] reported that muscle protein degradation signals in plasma increased in piglets with experimentally induced inflammation via lipopolysaccharide injections. Orellana et al. [44] also investigated the AA concentrations in plasma and found an increase in proline.

### 4.3. Limitations

A number of limitations of the present study must be noted. First, we introduced a relatively small number of farms (two farms/group). Although our results seem plausible to explain the relationship between blood metabolites and reproductive performance, a larger scale study was ideal and obviously needed. Therefore, at present, we are planning a large-scale study to confirm the results obtained in the present work. Second, we used sows from commercial farms to analyze parameters in feces and plasma, but the feed composition of each farm could not be obtained. However, all farms used general commercial diets, therefore we reasonably presume that differences in diet compositions were minimal.

## 5. Conclusions

In conclusion, blood metabolites, especially gut microbiota-derived metabolites, seemed to be associated with sow reproductive performance. Among the metabolites, harmful metabolites such as conjugates of IPMs and TMAO were apparently of more importance than health-conferring metabolites such as SCFAs in assessing the performance in sow reproduction by peripheral plasma. Based on the results of the present study, using plasma concentrations of *p*-cresyl sulfate and glucuronide, TMAO, potassium and TNF-alpha as potentially useful physiological indicators, a combinational evaluation can be carried out to further understand the sow reproductive performance.

## Figures and Tables

**Table 1:** Number of piglets weaned per sow per year from the four farms used in the study.

Farms	Number of Piglets Weaned/Sow/Year	Sampling Number
H1	29.4	10
H2	28.9	10
L1	19.5	10
L2	23.0	7

**Table 2:** Bacterial metabolites in feces.

Parameters		H Group	L Group	*p* Value
Organic acid (mmol/kg)	Acetate	67.8 ± 3.7	62.7 ± 3.0	0.30
	Propionate	22.9 ± 2.0	23.3 ± 1.4	0.87
	iso-Butyrate	2.8 ± 0.1	4.9 ± 0.6	0.004
	n-Butyrate	6.8 ± 0.7	7.6 ± 0.8	0.45
	iso-Valerate	3.5 ± 0.2	3.2 ± 0.3	0.40
	n-Valerate	0.6 ± 0.2	0.5 ± 0.2	0.61
Intestinal putrefactive metabolites (µmol/kg)	Phenol	1.8 ± 0.5	3.1 ± 0.6	0.08
	*p*-Cresol	929.2 ± 61.3	835.2 ± 86.1	0.37
	Indole	44.1 ± 3.2	33.9 ± 2.9	0.03
	Skatole	132.3 ± 15.4	95.8 ± 13.1	0.09

Succinate, lactate, formate and trimethylamine were not detected or detected only in traces in all feces; hence, they are omitted. Values represent the means (H group, n = 20; L group, n = 17) with standard error.

**Table 3:** Bacterial metabolites in plasma.

Parameters		H Group	L Group	*p* Value
Short-chain fatty acids (µmol/L)	Acetate	53.0 ± 9.7	87.1 ± 13.4	0.04
	iso-Butyrate	4.1 ± 0.5	5.1 ± 0.8	0.26
Glucuronyl- and sulpho-conjugates of intestinal putrefactive metabolites (µmol/L)	Phenyl glucuronide	0.00 ± 0.00	0.14 ± 0.05	0.02
	*p*-Cresyl glucuronide	8.9 ± 0.5	15.2 ± 1.0	<0.0001
	Phenyl sulfate	0.7 ± 0.1	1.0 ± 0.1	0.13
	Indoxyl sulfate	17.8 ± 1.4	18.4 ± 2.9	0.85
	*p*-Cresyl sulfate	3.7 ± 0.4	6.8 ± 0.8	0.002
Trimethylamine *N*-oxide (µmol/L)		5.2 ± 0.9	13.4 ± 2.3	0.003

Propionate, n-butyrate, n-valerate, iso-valerate, caproate and indoxyl glucuronide concentrations were not detected or detected only in one to four sows (total: 37 sows); hence, they are omitted. Values represent the means (H group, n = 20; L group, n = 17) with standard error.

**Table 4:** Biochemical parameters analyzed in blood plasma.

Parameters	H Group	L Group	*p* Value
Tumor necrosis factor-alpha (pg/mL)	37.1 ± 3.5	87.4 ± 17.8	0.01
Alanine aminotransferase (U/L)	39.1 ± 1.6	34.4 ± 1.7	0.051
Aspartate aminotransferase (U/L)	42.7 ± 2.5	61.2 ± 12.8	0.17
Creatine phosphokinase (U/L)	1407 ± 149	4604 ± 1698	0.08
Urea nitrogen (mg/dL)	9.6 ± 0.4	10.5 ± 0.6	0.19
Total cholesterol (mg/dL)	59.6 ± 2.0	60.3 ± 2.2	0.81
Creatinine (mg/dL)	2.22 ± 0.10	2.41 ± 0.11	0.22
Sodium (mEq/L)	143.3 ± 0.6	142.6 ± 1.0	0.51
Calcium (mg/dL)	10.3 ± 0.1	10.1 ± 0.1	0.07
Chloride (mEq/L)	103.7 ± 0.6	103.7 ± 0.7	0.99
Potassium (mEq/L)	4.8 ± 0.1	6.1 ± 0.5	0.02

Values represent the means (H group, n = 20; L group, n = 17) ± standard errors.

**Table 5:** Amino acid concentration in plasma (µmol/L).

Parameters	H Group	L Group	*p* Value
Tryptophan	86.0 ± 2.5	71.5 ± 3.1	0.001
Phenylalanine	101.3 ± 2.3	82.8 ± 3.1	<0.0001
Tyrosine	77.4 ± 2.6	82.1 ± 7.8	0.58
Leucine	205.4 ± 3.8	207.3 ± 8.7	0.85
Isoleucine	107.5 ± 2.8	104.7 ± 7.5	0.74
Valine	315.4 ± 7.5	293.5 ± 18.5	0.28
Methionine	45.6 ± 1.8	45.8 ± 2.8	0.95
Glutamic Acid	181.9 ± 6.8	192.3 ± 19.7	0.62
Proline	319.5 ± 11.5	361.4 ± 16.3	0.04
Threonine	204.8 ± 6.1	179.6 ± 16.1	0.16
Alanine	589.6 ± 23.1	546.1 ± 20.1	0.17
Serine	162.3 ± 6.3	158.9 ± 8.2	0.75
Glutamine	379.4 ± 10.4	334.6 ± 18.0	0.03
Glycine	1219.8 ± 77.4	1132.9 ± 86.2	0.46
Asparagine	52.8 ± 2.4	42.3 ± 2.8	0.01
Histidine	146.4 ± 9.0	111.9 ± 9.0	0.01
Lysine	207.1 ± 11.5	171.3 ± 10.0	0.03
Arginine	198.3 ± 7.5	195.8 ± 13.6	0.87

Values represent the means (H group, n = 20; L group, n = 17) ± standard errors.

## Data Availability

The raw data supporting the conclusions of this article will be made available by the authors on request.

## Supplementary Materials

The following supporting information can be downloaded at: https://www.mdpi.com/article/10.3390/metabo15110683/s1, Table S1: Instrument settings for the analysis of amino acid concentrations. Figure S1: The correlation coefficients between the fecal and plasma metabolites.

## Abbreviations

The following abbreviations are used in this manuscript:

AA	Amino acid
ALT	Alanine aminotransferase
AST	Aspartate aminotransferase
bCAA	Branched-chain amino acids
BUN	Urea nitrogen
CPK	Creatine phosphokinase
GC-MS	Gas chromatography–mass spectrometry
Group H	High reproductive performance group
Group L	Low reproductive performance group
IPM	Intestinal putrefactive metabolite
LC-MS/MS	Ultra-performance liquid chromatography tandem mass spectrometry
SCFA	Short-chain fatty acid
Tcho	Total cholesterol
TMA	Trimethylamine
d_9_-TMA	Trimethylamine-d_9_ hydrochloride
TMAO	Trimethylamine *N*-oxide
d_9_-TMAO	Timethylamine-d_9_ *N*-oxide
TNF	Tumor necrosis factor
