# Supplementary material for: Investigation of Metabolites in Feces and Plasma Associated with the Number of Piglets Weaned per Sow per Year
Source: Metabolites. 2025 Oct 22;15(11):683. doi: 10.3390/metabo15110683 (PMC12654331; doi:10.3390/metabo15110683)
Supplement: Supplementary file 1 [file metabolites-15-00683-s001.zip › metabolites-3825936-supplementary.pdf]

Supplementary Table S1. Instrument settings for the analysis of amino acid concentrations

Amino acid	Q1 (*m/z*)	Cone voltage (V)	Q3 (*m/z*)	Collision energy (V)
Tryptophan	205.2	18	188.1	10
Tryptophan ($^{13}C_{11}{}^{15}N_2$)	218.2	18	200.1	10
Phenylalanine	166.2	20	120.1	12
Phenylalanine ($^{13}C_9{}^{15}N_1$)	176.2	20	129.1	12
Tyrosine	182.1	20	136.1	12
Tyrosine ($^{13}C_9{}^{15}N_1$)	192.1	20	145.1	12
Methionine	150.2	18	56.0	18
Methionine ($^{13}C_5{}^{15}N_1$)	156.2	18	60.1	18
Leucine	132.2	18	86.1	10
Leucine ($^{13}C_6{}^{15}N_1$)	139.2	18	92.1	10
Isoleucine	132.2	18	86.1	10
Isoleucine ($^{13}C_6{}^{15}N_1$)	139.2	18	92.1	10
Proline	116.1	26	70.1	12
Proline ($^{13}C_5{}^{15}N_1$)	122.1	26	75.1	12
Valine	118.1	18	72.1	10
Valine ($^{13}C_5{}^{15}N_1$)	124.1	18	77.1	10
Glutamic acid	148.1	18	84.1	16
Glutamic acid ($^{13}C_5{}^{15}N_1$)	154.1	18	89.1	16
Threonine	120.1	40	74.0	20
Threonine ($^{13}C_4{}^{15}N_1$)	125.1	40	78.1	20
Aspartic acid	134.1	18	74.0	12
Aspartic acid ($^{13}C_4{}^{15}N_1$)	139.1	18	77.1	12
Alanine	90.0	18	44.1	8
Alanine ($^{13}C_3{}^{15}N_1$)	94.0	18	47.1	8
Serine	106.2	18	60.0	8
Serine ($^{13}C_3{}^{15}N_1$)	110.2	18	63.2	8
Glycine	76.1	18	30.1	8
Glycine ($^{13}C_2{}^{15}N_1$)	79.1	18	32.0	8
Glutamine	147.2	18	84.1	16
Glutamine ($^{13}C_5{}^{15}N_2$)	154.2	18	89.2	16
Asparagine	133.1	18	74.1	12
Asparagine ($^{13}C_4{}^{15}N_2$)	139.1	18	77.1	12
Histidine	156.2	25	110.1	16
Histidine ($^{13}C_6{}^{15}N_3$)	165.2	25	118.1	16
Lysine	147.2	18	84.1	18
Lysine ($^{13}C_6{}^{15}N_2$)	155.2	18	90.1	18
Arginine	175.2	27	70.1	22
Arginine ($^{13}C_6{}^{15}N_4$)	185.2	27	75.1	22

Cysteine was not detected using these settings.

Figure S1. The correlation coefficients between the fecal and plasma metabolites.

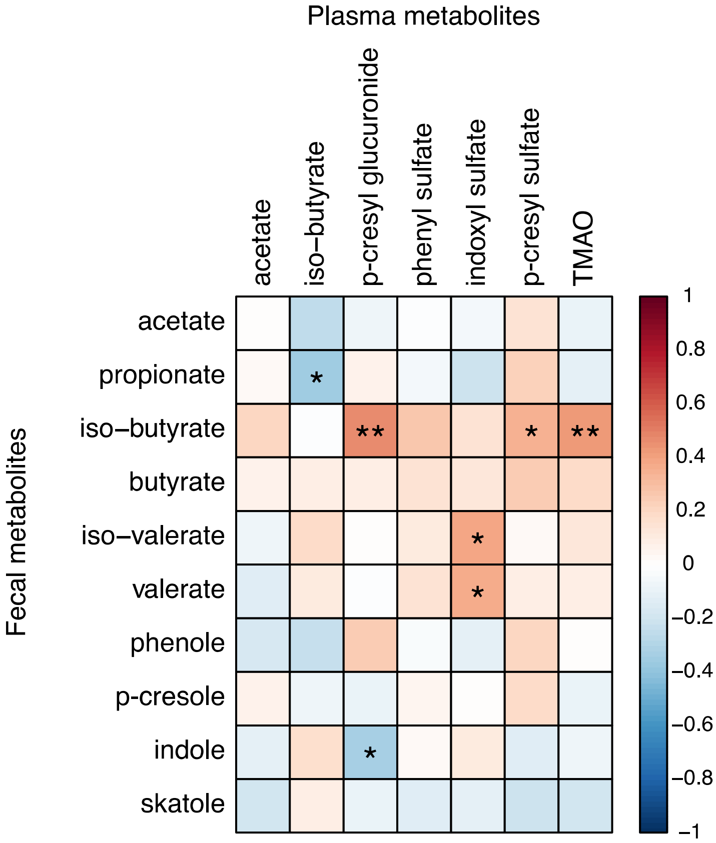

Asterisks indicate significant interactions between the parameters (*$P < 0.05$; **$P < 0.01$).
